# Cold Plasma Therapy in Chronic Wounds—A Multicenter, Randomized Controlled Clinical Trial (Plasma on Chronic Wounds for Epidermal Regeneration Study): Preliminary Results

**DOI:** 10.3390/jcm12155121

**Published:** 2023-08-04

**Authors:** Nessr Abu Rached, Susanne Kley, Martin Storck, Thomas Meyer, Markus Stücker

**Affiliations:** 1 Department of Dermatology, Venereology and Allergology, Ruhr-University Bochum, Gudrunstr. 56, 44791 Bochum, Germany; thomas.meyer@kklbo.de; 2 Scientific Institute for Health Economics and Health Research, Markt 9, 04109 Leipzig, Germany; susanne.kley@wig2.de; 3 Municipal Hospital Karlsruhe gGmbH, Moltkestraße 90, 76133 Karlsruhe, Germany; martin.storck@klinikum-karlsruhe.de

**Keywords:** plasma therapy, cold plasma, chronic wounds, wound-healing disorder, wound, diabetes mellitus, epidermal regeneration, health economics, plasma

## Abstract

Chronic wounds (CWs) pose a significant health challenge in clinical practice. Standard wound therapy (SWT) is currently considered the gold standard. However, recent evidence suggests that cold plasma therapy (CPT) holds promise for improving CWs. In light of this, the POWER study was conducted as a multicenter, randomized clinical trial to investigate the effect of large-area plasma application compared with SWT in patients with chronic, non-healing arterial or venous wounds on the lower leg. To analyze the interim results, we employed a comprehensive range of statistical tests, including both parametric and non-parametric methods, as well as GLS model regression and an ordinal mixed model. Our findings clearly demonstrate that CPT therapy significantly accelerates wound closure compared with SWT. In fact, complete wound closure was exclusively observed in the CPT group during the intervention period. Additionally, the CPT group required significantly less antibiotic therapy (4%) compared with the SWT group (23%). Furthermore, CPT led to a significant reduction in wound pain and improved quality of life compared with SWT. In conclusion, the study highlights that the combination of CPT and SWT surpasses monotherapy with SWT alone.

## 1. Introduction

Chronic wounds (CW) present significant medical and financial challenges for both patients and the healthcare system [1]. Meta-analyses have indicated a current prevalence of 2.21 chronic wounds per 1000 population, emphasizing its status as a common disease [2]. The German Initiative for Chronic Wounds e.V. and the German Society for Wound Healing and Wound Treatment e.V. define CWs as wounds that fail to heal within eight weeks [3,4]. Several known risk factors for CWs include diabetes mellitus, polyneuropathy, malnutrition, arterial circulatory disorders, chronic venous insufficiency, advanced age, and limited mobility [5]. CWs have a profound emotional, functional, and physical impact on patients, resulting in a strong desire for improved CW therapies [6].

Standard wound therapy (SWT), which involves guideline-based treatment of the underlying disease and primary wound care, represents a crucial component of CW treatment [4]. SWT comprises surgical debridement to remove necrotic tissues, antiseptic wound cleansing, the application of specialized dressings, and regular dressing changes. As the demand for outpatient wound therapies continues to rise, there is a need for further development and simplification of these treatments. In recent years, the investigation of cold plasma therapy (CPT) for chronic wounds has gained increasing attention. CPT has been explored in various conditions, including acute wounds, chronic wounds, pyoderma gangraenosum, dentistry, and basic oncology research [7,8,9].

CPT, a novel medical approach, uses cold plasma generated through the partial ionization of ambient air [10]. This generated plasma exerts antibacterial, antiviral, anti-inflammatory effects via diverse physical mechanisms, ultimately enhancing wound healing [11,12,13,14]. Moreover, studies have demonstrated that plasma promotes angiogenesis, facilitating the formation of new blood vessels, which further enhances wound healing [15]. Guo et al. conducted a meta-analysis on CWs, reporting that CPT therapy was more effective in reducing wound area compared with SWT [16]. However, certain types of plasma devices have received critical appraisal, indicating that they have failed to meet expectations [17].

In contrast to previous plasma application techniques, the applicator used in our study generates a homogeneous plasma field over a large treatment area (11 × 11 cm) and achieves optimal treatment in just two minutes, regardless of wound size or depth. Most previous plasma therapies have been limited to much smaller areas. Compared to other devices, the system used in this study ensures treatment consistency and reproducibility as the plasma application is automated, eliminating examiner variability. The Plasma On Chronic Wounds for Epidermal Regeneration (POWER) study aimed to compare CPT with the gold standard SWT, particularly assessing the effect of CPT on wound healing in a realistic set up. Consequently, patient selection was not biased toward favorable cases, and exclusion criteria were minimized to demonstrate a clinically significant effect applicable to real-world clinical practice.

## 2. Materials and Methods

### 2.1. Study Design

The POWER study is a multicenter, randomized clinical trial (RCT) designed to investigate the effect of cold plasma therapy (CPT) in chronic, non-healing wounds. The trial began in 2021 and is expected to be completed by the end of 2024. The study compared the outcomes of the CPT group and the standard wound therapy (SWT) group to evaluate treatment differences over a period of four weeks with three visits per week. Participants in both groups were randomly recruited from multiple centers.

The primary endpoint of the study was the relative reduction of the wound area after the four-week treatment period. In this study, the wound area is digitally determined planimetrically at each study visit using the SilhouetteStar^®^ camera (medical device class I, Aranz medical, Christchurch, New Zealand). Secondary endpoints included events of complete wound closure, changes in wound tissue quality, bacterial load, occurrence of wound relapse, wound pain, quality of life, and necessity of hospitalization. Pain levels were assessed using the numeric rating scale (NRS) [18]. Wound closure was defined as 100% epithelialization of the wound without the need for wound drainage.

In the intervention group, patients received wound therapy according to the current guideline in addition to CPT for four weeks [4]. A single CPT session lasted for 2 min and was performed three times a week, resulting in a total of 12 treatments. The control group received standard wound therapy according to the current guideline without CPT. The investigators received training on guideline-based therapy for SWT prior to the study and decided at each visit whether wound debridement was necessary. Only trained investigators who were approved by the ethics committee could administer the intervention. Follow-up visits were scheduled at 3 and 6 months after the initial treatment.

Block randomization was employed with an allocation ratio of 1:1 to ensure a balance between the treatment groups. Blinding both the patients and investigators was not feasible due to technical constraints. This study is registered as a prospective study in the German Clinical Trials Register (DRKS00019943).

### 2.2. Patients

The study included patients who were at least 18 years old and met specific criteria. Chronic wounds on the lower leg that were not acutely infected and did not require antibiotic treatment at baseline were included. To be eligible for participation, patients had to have a wound size ranging from 5 to 100 cm^2^ and a chronic arterial or venous wound on the lower leg that had persisted for at least 8 weeks.

Certain exclusion criteria were applied, such as the presence of a pacemaker or defibrillator, pregnancy or breastfeeding, chronic infectious diseases, tumor diseases, immunosuppression, the need for revascularization due to peripheral occlusive disease, and renal insufficiency requiring dialysis. Additionally, wounds with mixed causes were excluded from the study, and only wounds with a clearly defined arterial or venous cause were included.

### 2.3. CPT Device

The CPT device used in the study was the CPTcube/CPTpatch system, developed by Coldplasmatech GmbH, Greifswald, Germany. This system consists of two main components: the CPTcube, which is a cubic device with a fully automated treatment program, and the CPTpatch, which is a thin plasma-generating foil dressing used for wound sealing and plasma application. As a system suitable for treating secondary healing wounds, it is classified as IIb.

The CPTpatch is placed above the wound and is capable of detecting all relevant treatment parameters to ensure optimal treatment conditions within a 2 min treatment session, regardless of the size and shape/depth of the wound.

The technology employed in the CPT device is a surface dielectric barrier discharge (DBD). This setup offers several advantages, including independence from noble gases, the absence of additional electrodes, and the elimination of the need for a human counter electrode.

For further technical details about the CPT device, please refer to Appendix A and Appendix B. Further information can be found under supplementary materials.

### 2.4. Statistical Analysis

For the interim analysis of the primary outcome, data were available for 48 patients. One patient (CPT group, 57 years of age, male, no diabetes mellitus) had no wound photographs up to day 27. The intention to treat (ITT) principle was used for this analysis to avoid bias. A sample size calculation showed that 134 patients are needed for the results. It has been specified in the study protocol that an interim analysis will be performed after 1/3 of the calculated sample size (number of sample size required for the interim analysis; n = 45). Normal distribution was tested with the Shapiro–Wilk test and the Q-Q diagram. To detect differences, we used the chi-square test, the t-test for parametric values, and the Mann–Whitney U test for nonparametric values. To optimally handle unequal time intervals, imbalanced data, and missing values, a popular full likelihood approach, namely generalized least squares (GLS), is used to model longitudinal responses of the continuous primary outcome [19,20]. Addressing model assumptions, including appropriate correlations between measurements on the same patient and homoscedasticity of residuals, is important for optimal model fit and honest inference [19], as emphasized in the 2016 American Statistical Association statement [21,22]. Besides knowledge of the model assumptions in order of importance, diagnostic tools that can be used to assess whether some of these assumptions are reasonable and dealing with violated assumptions are crucial for modeling [23]. The steps of model diagnostics, however, are not straightforward and can be cyclical for longitudinal studies [24], similar to some diagnostics in medicine. Moreover, background knowledge to guide some decisions in modeling is desirable, but rarely supported by the medical literature, e.g., correlation structures and transformations. Using general scientific knowledge, we prespecified a seemingly plausible correlation structure for visits (autoregressive of order 1 for continuous days since baseline visit) and assumed homoscedasticity for the initial GLS model in the statistical analysis plan. In this initial GLS model, treatment is included by the factor treatment and the interaction between treatment and visit and adjusted for the baseline values of the primary outcome, diabetes mellitus, age, gender, and study center [19]. Departures from the linearity of continuous variables, including visits and baseline values of the primary outcome, are modeled with restricted cubic splines to address a key assumption of the regression framework [19]. Departures from the linearity of continuous variables, including visits and baseline values of the primary outcome, are modeled with restricted cubic splines to address a key assumption of the regression framework [19].

We present the results of a planned interim analysis including visits up to day 27 for which linearity was prespecified to avoid overfitting, which may be justified in this setting [25]. Nevertheless, we checked the assumptions of linearity, additivity, appropriate correlation structure, and homoscedasticity, and examined outliers for measurement or data entry errors using diagnostic tools such as semivariograms, linear regression smoother loess, residual plots, information criteria, and Box–Cox transformation [19,20,26].

To correct for violated assumptions, we used nugget effects for correlation structure, as often required [20,27], and either weighting or transformation to achieve the homoscedasticity of residuals [20,26]. For interpretability, we prefer the original scale to transformations (unless the logarithmic transformation would have been appropriate) [28].

The physical and mental scores of the SF-12 questionnaire were treated as continuous variables, as suggested by Morfeld et al. [29]. To analyze the treatment effect on these scores, we employed a simple linear model (ordinary least squares) that adjusted for covariates, including the baseline score, the exact day of the final examination, age, gender, site, and diabetes mellitus. Further statistical explanations of physical and mental scores can be found in Appendix C.

The statistical analyses were performed using SAS software, version 9.4 (SAS Institute, Cary, NC, USA) and R software (R Core Team, version 4.3.1, Vienna, Austria, 2022). Specific packages such as rms [19], nlme [20], and applications of ggplot2 [30] were utilized. A significance level of *p* < 0.05 was considered statistically significant.

## 3. Results

Data for the interim analysis of the primary outcome were available for 48 patients. However, one patient in the CPT group, a 57-year-old male without diabetes mellitus, did not have wound photographs up to day 27. To minimize bias, the analysis was conducted according to the intention-to-treat (ITT) principle. A sample size calculation determined that 134 patients are required to obtain statistically significant results. The study protocol specified that an interim analysis would be performed after one-third of the calculated sample size was reached, which corresponds to a sample size of 45.

### 3.1. Baseline Characteristics

Table 1 presents the baseline characteristics of the study population. The majority of patients were male (62%) compared to female (38%). The mean age ± SD was 69.5 ± 11.3 years, and the median BMI was 30.9 kg/m^2^ (interquartile range, IQR, 26.9–35.1). Approximately 28% (n = 13) of patients had diabetes mellitus as a comorbidity. There were no significant differences between the two groups regarding gender distribution, BMI, wound type, and wound area at baseline (*p* = 0.8, >0.9, 0.14, and 0.4, respectively).

In the CPT group, the mean age ± SD was 72.2 ± 10.9 years, while in the SWT group, it was 66.5 ± 11.2 years. Approximately 40% (n = 10) of patients in the CPT group had diabetes mellitus as a comorbidity, compared to 14% (n = 3) in the SWT group. Although patients in the CPT group were older and had a higher prevalence of diabetes mellitus, these differences were not statistically significant (*p* = 0.1 and 0.08, respectively).

To ensure better comparability of comorbidities, the Charlson Comorbidity Index (CCI) was calculated [31]. The CCI scores did not significantly differ between the two groups, indicating that comorbidities did not influence the study outcomes (*p* = 0.5).

### 3.2. Reduction of Wound Size

**Figure 1:**
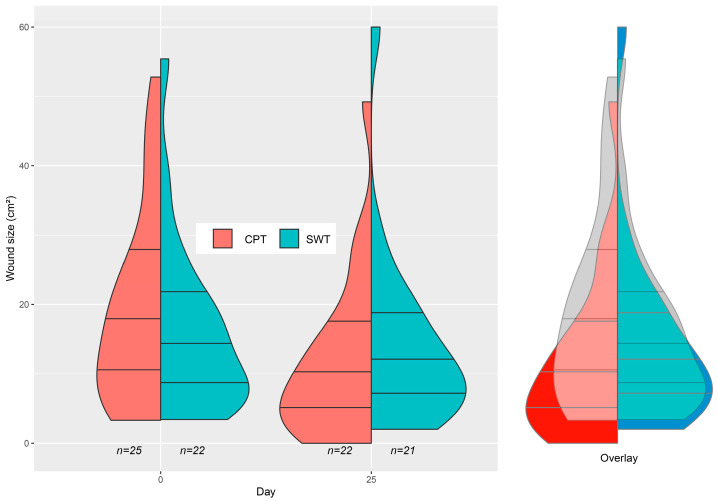
Violin plots with densities of the wound sizes (first horizontal line, first quartile; second line, median, and third line, third quartile); left plot on day 0, middle plot on day 25, and right plot as overlay of day 0 and 25 (gray area represents the data from day 0 in the overlay); CPT, cold plasma therapy; SWT, standard wound therapy.

Figure 1 displays a violin plot comparing baseline wound size to day 25 ± 2, providing a general overview of the treatment progression in both groups. The varying shapes on day 25 visualize the favorable effect of the CPT group, most pronounced near complete wound healing. As per the study protocol, the last visit occurred on day 25 ± 2. In cases where data were missing on day 25, the mean value from days 24 and 26 was used. If data were still missing, the mean value from days 23 and 27 was used. Patients without any visits between days 23 and 27 were not included in the violin plot to prevent distortions resulting from missing treatment data. However, these patients were integrated into the model calculations and could be evaluated.

Table 2 summarizes the reduction in wound size for each group and highlights the more favorable effect of CPT. In the CPT group, 16% of wounds achieved more than 90% closure, including complete wound closure, while no such closures were observed in the SWT group (0%). Furthermore, 28% of wounds in the CPT group exhibited a reduction of at least 60%, which was not observed in the SWT group (0%). A reduction in wound area of at least 40% was observed in 40% of the CPT group and 18% of the SWT group. Moreover, 56% of wounds in the CPT group and 27% of wounds in the SWT group experienced a decrease in size of at least 25% during the one-month intervention.

**Figure 2:**
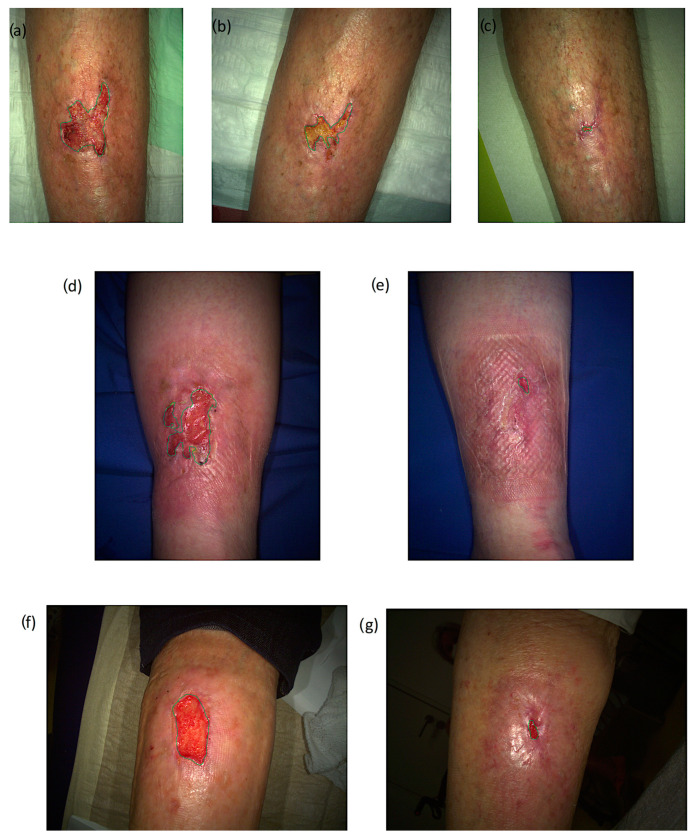
Picture series of the wound healing with CPT treatment. (**a**–**d**): A 79-year-old patient with a BMI of 23.9 kg/m^2^ and a wound area of 7.2 cm^2^ at baseline received wound therapy with CPT, (**a**) before therapy initiation; (**b**) after 4 weeks with 70% reduction of the wound area; (**c**) after 12 weeks follow-up with 99% reduction of the wound area. (**d**,**e**): A 67-year-old patient with a BMI of 37.9 kg/m^2^ and a wound area of 7.1 cm^2^ at baseline; (**e**) before therapy initiation; (**f**) after 4 weeks with 99% reduction of the wound area. (**f**,**g**): A 83-year-old patient with a BMI of 22.4 kg/m^2^ and a wound area of 7.1 cm^2^ at baseline; (**e**) before therapy initiation; (**f**) after 4 weeks with 95% reduction of the wound area.

Figure 2 presents a series of wound photographs from three patients treated with CPT (Figure 2a–g). These clinical case examples demonstrate the effectiveness of CPT for treating CW.

### 3.3. GLS Model Regression for Evaluating Wound Closure

**Figure 3:**
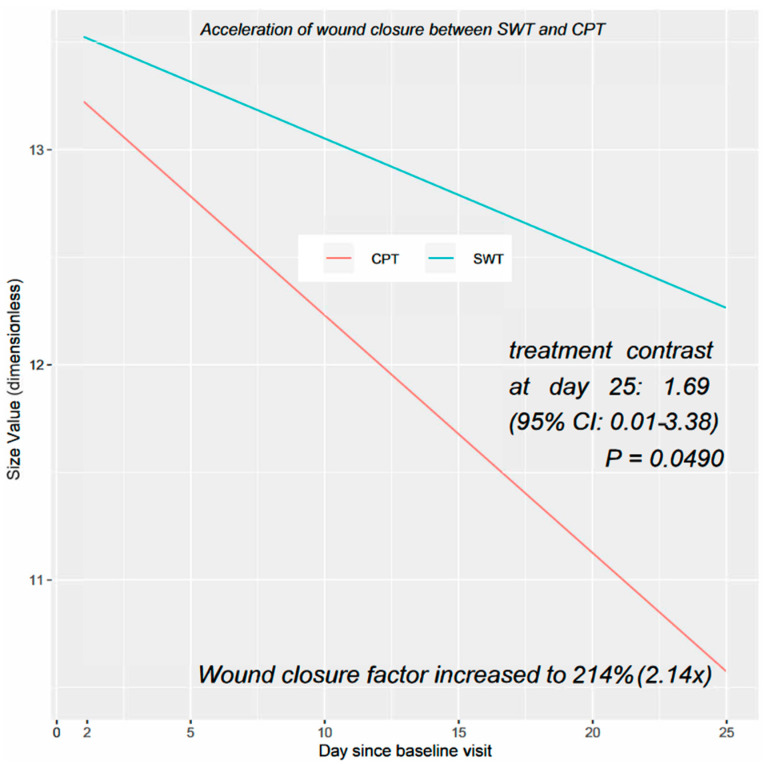
Wound size in treatment groups up to 25 days in the final GLS model adjusted for gender, age, baseline value, diabetes mellitus, and study site; CPT, cold plasma therapy; SWT, standard wound therapy.

In the GLS regression analysis, a total of 474 follow-up visits in 47 patients were included, starting from the predefined initial model. The model was adjusted for gender, age, baseline value, diabetes mellitus, and study site to provide a comprehensive evaluation of the results. The final GLS model and the square root transformed model both met the assumptions of the model well and exhibited similar treatment patterns over the 25-day period, with the treatment difference gap increasing with each day (see Figure 3 for the final model).

On day 25, the CPT group demonstrated a significantly superior performance compared to the SWT group (treatment contrast for the final model; *p* = 0.0394 for the transformed model). The increased rate of wound closure was noticeable as early as the first treatment with CPT (day 2) and continued to improve steadily until day 25. In comparison to standard wound therapy, the rate of wound closure in the CPT group increased to 214%, corresponding to a factor of 2.14.

The final syntax of the model and additional information can be found in Appendix D.

### 3.4. Ordinal Mixed Model for Evaluating Wound Pain

In the analysis of pain, a total of 489 follow-up observations were available for 48 patients, considering both active and passive pain. For active pain, the joint test for treatment and the interaction between treatment and visit resulted in a *p*-value of 0.1255, indicating no significant difference between the treatment groups. However, for passive pain, the corresponding *p*-value was 0.0003 (*p*-value = 0.0120 for the interaction), favoring the CPT group.

**Figure 4:**
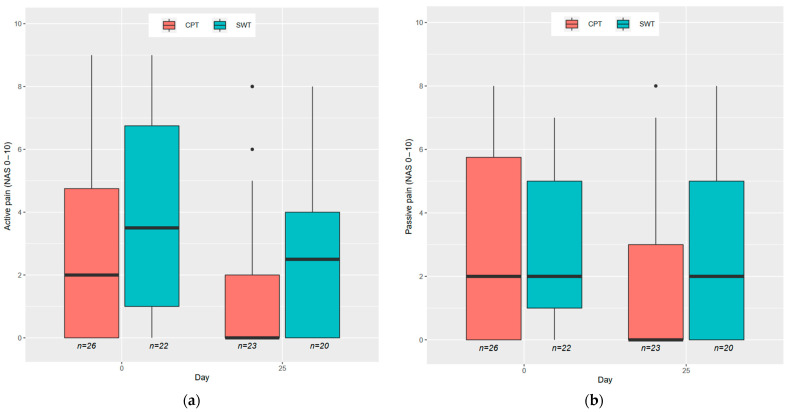
Box plots with distribution of pain levels for CPT and SWT in the numeric rating scale (NRS). Time 0 before the intervention, day 25 for the end of the intervention: (**a**) active pain; (**b**) passive pain. Passive pain differed significantly between day 0 and day 25 in the CPT group (*p* = 0.0001).

By day 25, the difference between the groups was most pronounced in terms of the outcome level 0, indicating the absence of passive pain (*p* < 0.0001; *p* = 0.018 for the joint test of the 10 pain levels observed, ranging from 0 to 10; see Figure 4). Compared to the SWT group, the probability of a patient experiencing no pain (level 0) increased to a greater extent in the CPT group during therapy.

For further details regarding the statistical analysis, please refer to Appendix E.

### 3.5. Frequency Analysis of Antibiotic Therapies in the Groups

The analysis considered the period between the inclusion visit and the achievement of the maximum treatment and the last visit of the intervention (EMB), which corresponds to a total of 32 days since an EMB was performed on day 32.

During this period, a total of 6 patients required the administration of antibiotics, including 5 of the 22 patients (23%) in the SWT group and 1 of the 26 patients (4%) in the CPT group. Overall, there was a higher incidence of antibiotic use in the SWT group compared to the CPT group.

When analyzing the period under consideration, the Pearson chi-square test demonstrated a significant difference in the administration of antibiotics, favoring the CPT group (*p*-value = 0.049).

This finding suggests that the CPT group required significantly fewer instances of antibiotic therapy compared to the SWT group.

### 3.6. Quality of Life (QoL)

**Figure 5:**
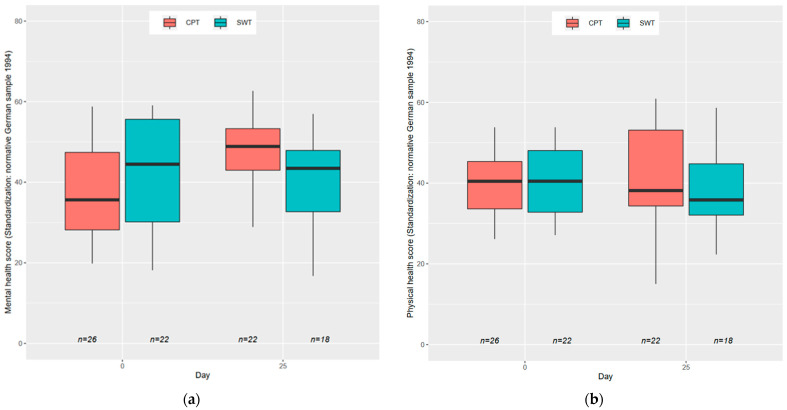
Box plots with mental and physical health score: (**a**) mental health score (standardization: normative German sample 1994); (**b**) physical health score (standardization: normative German sample 1994). On day 25, CPT outperformed the SWT group in quality of life (*p* = 0.0001).

Figure 5 demonstrates a substantial improvement of quality of life (QoL) in the CPT group, while no such improvement is observed in the SWT group. At the final visit on day 25, CPT outperformed SWT with a treatment contrast of −10.22 (95% CI: −5.46 to −14.97, *p* = 0.0001). The mean score for the CPT group was 50.72 (95% CI: 46.51–54.93), whereas the mean score for the SWT group was 40.50 (95% CI: 36.22–44.79). Sensitivity analyses were performed to address overfitting concerns, and the superiority of CPT over SWT at the final visit remained unquestioned. Importantly, the observed difference of one standard deviation is considered an important and clinically meaningful improvement. Analysis of physical scores from the SF-12 revealed no significant treatment contrasts (*p* = 0.5150) in the analyzed sample size.

For transparency, the data for the normative US sample from 1990 is available in the supporting material (Appendix F).

## 4. Discussion

CWs are defined as wounds that have not healed within 8 weeks [3]. There are various underlying causes of CWs, and the inflammatory environment, characterized by altered cytokine and growth factor profiles, plays a crucial role [32]. New therapies, such as CPT therapy, aim to eliminate the inflammatory milieu [32]. CWs are often colonized by bacteria, which can impede wound healing and promote the release of pro-inflammatory cytokines [33]. Several studies have demonstrated the antibacterial and anti-inflammatory effects of CPT in inflammatory diseases [34,35,36], including CW treatment [37,38,39]. Isbary et al. conducted an RCT using argon plasma and found a significant reduction in bacterial load in treated wounds, irrespective of the bacteria type [37]. In our study, we observed a significant reduction in antibiotic use with CPT therapy compared with SWT (*p* = 0.049). To the best of our knowledge, this is the first RCT demonstrating a reduction in antibiotic use with CPT therapy, which is crucial considering the rising problem of antibiotic resistance caused by excessive usage [40]. In CWs, antibiotics are often overused and indiscriminately prescribed, leading to increased antibiotic resistance [41], especially against beta-lactam antibiotics [42]. The antibacterial effect of CPT therapy could serve as a valuable alternative in CW, preserving antibiotics and helping to avoid antibiotic resistance in the future.

Additionally, cold plasma has been found to influence wound healing processes in CWs. In vivo and in vitro studies using mouse models have shown that cold plasma promotes the synthesis of collagen 1 and collagen 3, which are essential components of the dermis and connective tissue [43,44,45].

Several clinical trials have also reported the benefits of CPT on wound healing [46]. For instance, a randomized clinical trial on CPT in diabetic foot ulceration demonstrated a significant reduction in wound surface area and time to wound closure [46,47]. The median baseline wound area in the CPT group of Stratmann et al. was 2.82 cm^2^, which was smaller than the median in our study (17 cm^2^; ICR, 7.2–25.6). Another recent RCT reported a significantly higher complete wound healing rate with CPT compared to best practice in CWs (complete wound closure rate: 58.97% vs. 5.13%) [47]. In Strohal’s study, the median wound size was also smaller than in our study (3.52 cm^2^ vs. 17 cm^2^). Nevertheless, our RCT yielded similar results, with a significant 214% increase in the wound closure factor in the CPT group compared with SWT (*p* = 0.049). The treatment contrast at day 25 between the CPT and SWT groups was also significantly different (1.69, 95% CI 0.01–3.38; *p* = 0.049). However, the wide confidence interval is due to the smaller sample size of the interim analysis (n = 48) relative to the required sample size for the final analysis (n = 134). The faster closure of CW can reduce treatment time, pain, and costs, leading to significant cost savings for healthcare systems.

In contrast to these positive findings, a meta-analysis by Assadian et al. reported no significant benefit of cold plasma therapy on wound healing in CWs [17]. However, comparing cold plasma studies on wound healing is challenging due to differences in study designs, endpoints, and the physical properties of various devices [17]. While cold plasma did not demonstrate improved reduction in bioburden compared to controls [17], pooled data from the studies showed a reduction in bacterial load after CPT (56%) versus controls receiving SWT, which also included local antiseptics that significantly reduce bacterial growth (61%) [17]. Therefore, the results of the meta-analysis by Assadian et al. should be interpreted cautiously. Moreover, the application of handheld devices may vary depending on the user, leading to higher study heterogeneity and more variable results. In our RCT, we used a device that applies cold plasma independently of the user, ensuring depth- and size-independent treatment. The fixed closure between the wound and the CPT patch prevents plasma gas from escaping, resulting in more efficient treatment as the optimal treatment condition is consistently maintained. Generally speaking, to achieve optimal efficacy and comparability, it is crucial for the products to be easy to use and reproducible [48]. The device used in our study fulfilled the criterion of easy reproducibility as it operates automatically. These factors could explain the increased effectiveness of CPT therapy observed in our study compared with other research results.

It should be noted that patients in the CPT group were older and had a higher prevalence of diabetes mellitus compared with the SWT group (median age 72.2 vs. 65.5 years; diabetes mellitus rate 40% vs. 14%). However, these differences were not statistically significant (*p* = 0.1 and 0.08, respectively). It is possible that the effects of CPT in this study were underestimated. This hypothesis will be further explored in the final analysis.

CWs, particularly dressing changes, are often associated with severe pain, significantly impacting patients’ quality of life [49,50]. CPT therapy offers a safe and painless alternative for wound treatment [17]. No severe adverse events had occurred during the therapy. Our results demonstrate that CPT can effectively reduce wound pain, even reducing the pain score to zero on the numeric rating scale, which is a significant relief for patients. Additionally, it is expected that the use of CPT can lead to a decrease in the consumption of painkillers, thereby avoiding the potential adverse effects associated with their use. Further studies should be conducted to investigate analgesic consumption in CPT compared with SWT.

Until now, no results are known about the impact of CPT therapy on quality of life. In this study, CPT therapy was associated with significant QoL compared to the SWT group (*p* = 0.0001). Quality of life was assessed using the validated SF-12 questionnaire, which consists of two items [51]. The item Physical Component Summary score (PCS) provides information about physical functioning and pain. The Mental Health Component Summary score (MCS) presents the patient’s mental and spiritual health. CPT therapy mainly led to an improvement in the MCS. One possible explanation for the improvement in MCS could be the faster wound healing in the CPT group. In patients with faster wound closure, QoL may increase as patients perceive the treatment success themselves and this may lead to more satisfaction. Psychological stress can modulate wound healing indirectly through adopting health-damaging behaviors, so there is a link between wound healing and psyche [52]. Chronic pain is associated with structural and functional changes in the brain [53]. This demonstrates that the neural connection between pain and wound healing should not be underestimated. Furthermore, the reduction in passive pain could be associated with the increase in QoL. A reduction in passive pain leads to less fear avoidance [42], so that patients with CPT have more participation in life. The reduction of pain can also improve sleep patterns [43], which also affects QoL. CPT is not only a safe method, but also a method that improves QoL.

CWs pose a significant financial burden on the healthcare system due to the prolonged healing process, which can span several years. The treatment of CWs incurs substantial costs related to bandages, medical care, and hospitalization [37,38]. Strohal et al. suggest that the use of CPT in CWs may result in medico-economic benefits [35]. Therefore, investigating the economic aspects of CPT will be a focus of future studies.

The strength of our study lies in its robust design, which closely reflects the real-world conditions of CW patients. The study included wounds of various sizes, ranging from small to very large (up to 100 cm^2^), thereby capturing the true diversity encountered in clinical practice, rather than using an artificial study design. The intention-to-treat principle was employed for this analysis, ensuring that all patients were included as initially intended, thus minimizing the risk of biased results favoring one group over the other. Consequently, three patients with wounds smaller than 5 cm^2^ were included in the evaluation, despite the study protocol specifying a minimum size of 5 cm^2^. One limitation of the POWER study is the inability to blind the participants and investigators due to technical constraints.

In the interim analysis, it was not possible to determine how effective cold plasma is for arterial and venous wounds. Stratification with regard to the underlying disease (chronic venous insufficiency (CVI) or peripheral arterial disease (PAOD)) is performed per study protocol and will be presented at the end of the recruitment.

In conclusion, our study demonstrates the promising potential of CPT therapy, particularly for larger wounds, in significantly improving wound healing rates compared with SWT. The positive effects of CPT on wound healing, wound pain reduction, and antibiotic frequency have been confirmed. Importantly, this is the first RCT to employ CPT technology that works on large wound areas. The interim analysis provides valuable clinical insights, especially regarding antibiotic use during CPT. Additional data from the final analysis will further enhance our understanding of the study’s other endpoints.

## 5. Conclusions

In conclusion, the results of our interim analysis demonstrate that the combination of CPT and SWT is superior to SWT alone in terms of effectiveness. Specifically, CPT leads to a significant increase to over 210% in the wound closure factor compared with monotherapy with SWT. This indicates that wounds heal significantly faster under CPT treatment. Furthermore, CPT significantly reduces the need for antibiotic therapy, increases QoL, and provides a notable reduction in wound pain compared with the current gold standard in wound treatment. The user-independent application of cold plasma minimizes result variability and bias, highlighting its potential to establish a new standard in wound healing.

## Figures and Tables

**Table 1:** Personal and baseline characteristics of patients with primary outcome data from the POWER study.

Parameter	Overall	CPT	SWT	*p*-Value
	(n = 47)	(n = 25)	(n = 22)	
Gender, n (%)				
male	29 (62)	15 (60)	14 (64)	0.8
female	18 (38)	10 (40)	8 (36)	
Age, y				
mean (± SD)	69.5 (±11.3)	72.2 (±10.9)	66.5 (±11.2)	0.08
median (Q1, Q3)	70 (64, 77.5)	73 (69, 80)	66.5 (60, 72)	
median (range)	70 (45–87)	73 (45–87)	66.5 (46–86)	
BMI, kg/m^2^				
mean (± SD)	32.4 (±8.6)	32.2 (±8.4)	32.6 (±9)	>0.9
median (Q1, Q3)	30.9 (26.9, 35.1)	32.7 (25.6, 35.1)	30.5 (27.4–34.9)	
median (range)	30.9 (21.5–58.1)	32.7 (21.9–58)	30.5 (21.5–58.1)	
Baseline wound size, cm^2^				
mean (± SD)	17.3 (±13.2)	18.7 (±13.9)	15.7 (±12.5)	
median (Q1, Q3)	14.8 (7.1, 20.8)	17.0 (7.2, 25.6)	13.3 (6.28, 19.3)	0.4
median (range)	14.8 (3.30–55.4)	17.0 (3.3–52.8)	13.3 (3.4–55.4)	
Diabetes mellitus, n (%)				
no	34 (72)	15 (60)	19 (86)	0.1
yes	13 (28)	10 (40)	3 (14)	
Wound type, n (%)				
venous	39 (83)	18 (75)	21 (91)	0.14
arterial	8 (17)	6 (25)	2 (9)	
CCI score				
median (Q1, Q3)	3 (2,4)	3.5 (2,5)	3 (2,4)	0.5

n, absolute number of patients; %, relative number of patients; SD, standard deviation; Q1, first quartile; Q3, third quartile; y, years; BMI, body mass index; CPT, cold plasma therapy; SWT, standard wound therapy; CCI, Charlson comorbidity index.

**Table 2:** Reduction of the wound area during the four-week intervention.

Parameter	CPT (n = 25)	SWT (n = 22)
≥90% reduction of the wound area, n (%)	4 (16)	0 (0)
≥60% reduction of the wound area, n (%)	7 (28)	0 (0)
≥40% reduction of the wound area, n (%)	10 (40)	4 (18)
≥25% reduction of the wound area, n (%)	14 (56)	6 (27)

n, absolute number of patients; %, relative number of patients; CPT, cold plasma therapy; SWT, standard wound therapy.

**Figure A1:**
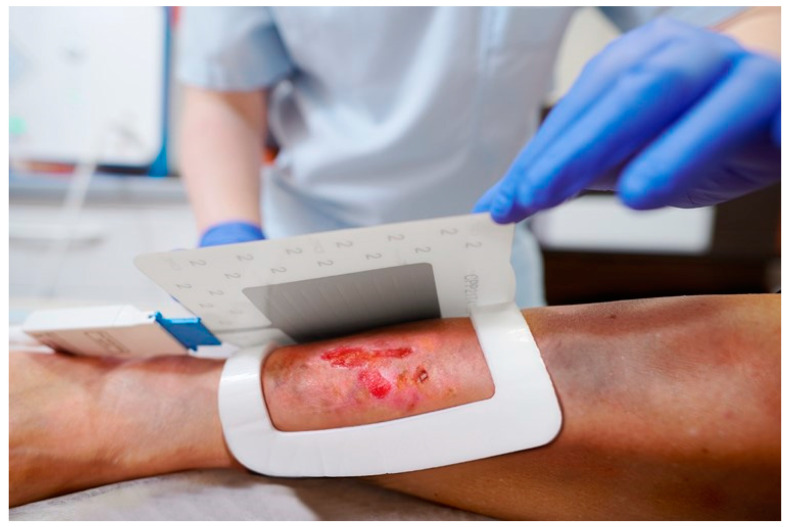
An exemplary picture that shows the application of CPT (CPTpatch) to the chronic wound.

**Figure A2:**
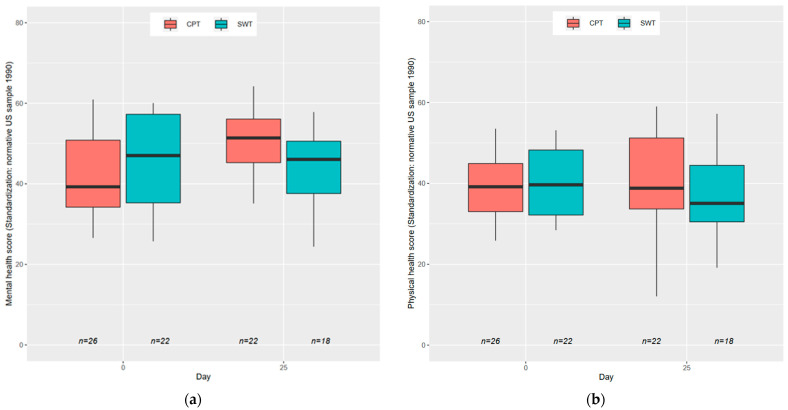
Box splots with mental and physical health score: (**a**) mental health score (standardization: normative US sample 1990); (**b**) physical health score (standardization: normative US sample 1990).

## Data Availability

The data presented in this study are available on request from the corresponding author. The data are not publicly available for privacy reasons. The full study protocol is available on request from the corresponding author.

## Supplementary Materials

The following supporting information can be downloaded at: https://www.mdpi.com/article/10.3390/jcm12155121/s1, The CONSORT 2010 checklist can be found completed under supplementary material. For statistical analysis, we used ggplot2 [28] which is available on https://stackoverflow.com/questions/47651868/split-violin-plot-with-ggplot2-with-quantiles/47652563#47652563, accessed on 5 May 2023.

## Appendix B

- Operating voltage: 7 kV
- Operating frequency: 6 kHz
- Operation in pulsed mode: duty cycle 10%
- Plasma power: about 10 W
- Treatment area: 11 cm × 11 cm = 121 cm^2^
- Treatment time fix: 2 min
- Operating gas for plasma: ambient air

## Appendix C

To analyze the treatment effect on the physical and mental scores of the SF-12 questionnaire, we employed a simple linear model (ordinary least squares) that adjusted for covariates, including the baseline score, the exact day of the final examination, age, gender, site, and diabetes mellitus. However, given the risk of overfitting due to the model’s 12 degrees of freedom and the limited number of observations (40), we took three approaches to address this issue and reduce the degrees of freedom. First, we adjusted the treatment effect solely for the baseline values. Second, to ensure an acceptable ratio of effective degrees of freedom to the number of observations [19], which should be better than 1:5, we penalized the full model, including treatment as the exposure of interest. Finally, we penalized the full model, excluding treatment, following the methodology proposed by Harrell [19]. Robust variances were used, as recommended by Harrell [19], to provide more reliable estimates

## Appendix D

Box A1 R syntax used for final model for evaluating wound closure.

R syntax
Initial model
gls(Area ~ treatment * visit + baseline + age + gender + site + diabetes, data = MyData, correlation =corCAR1(form = ~visit | patient ))
Final model
gls(Area ~ treatment * visit + baseline + age + gender + site + diabetes, data = MyData, correlation =corRatio(nugget=TRUE, form = ~visit | patient ), weights = varPower(form= ~baseline))
Square root model (sensitivity analysis)
gls(sqrt(Area) ~ treatment * visit + sqrt(baseline) + age + gender + site + diabetes, data = MyData, correlation =corRatio(nugget=TRUE, form = ~visit | patient ))
In R, “treatment * visit” means “treatment * visit + treatment + visit”.

## Appendix E

Because pain is ordinal, its median approaches zero during follow-up, and the distribution of the outcome is asymmetric, which introduces bias and inefficiency when using a metric model [54], an ordinal mixed model, specifically a random-intercept proportional odds model, was used [55]. The syntax of Stata software, release 17.0 (Stata Corporation, College Station, version 17.0, TX, USA) is shown in Box A2.

Box A2 Stata syntax for the random-intercept proportional odds model for evaluating wound pain.

Stata syntax
meologit pain i.treatment##c.visit pain0 age i.gender i.site i.diabetes ||patient:
